# Supplementary material for: Are carbon emissions trading and green financial instruments synergistic? -Comprehensive quantitative research based on content analysis
Source: PLoS One. 2024 Mar 7;19(3):e0298601. doi: 10.1371/journal.pone.0298601 (PMC10919601; doi:10.1371/journal.pone.0298601)
Supplement: S1 Data — (ZIP) [file pone.0298601.s001.zip › Data/地级市Z_F工作报告.zip!/║■─╧/┬a╡╫╩╨/└┤╫╘╣1⁄2╓┌║┼í╛┬φ┐╦╩2╛▌═°í┐_2008.docx]

各位代表：

　　现在，我代表市人民政府，向大会作工作报告，请予审查，并请各位政协委员和列席人员提出意见。

　　一、2007年政府工作回顾

　　2007年，全市上下以庆祝党的十七大胜利召开和娄底地级建制30周年为契机，深入贯彻科学发展观，全面落实省第九次党代会和市第三次党代会精神，按照“又好又快、后发赶超”的总体要求，着力深化改革开放，强力推进“三化”进程，努力促进社会和谐，全市经济社会平稳快速发展，较好地完成了市二届人大四次会议确定的各项目标任务。

　　经济发展提速增效。坚持以加速赶超为目标、以优化结构为手段、以项目建设为突破口，全市经济总量增加、速度加快、效益提升。全年完成生产总值437亿元(预计数，下同)，增长13.5%，超过预期目标1个百分点；完成地方财政总收入32亿元，增长25%，其中财政一般预算收入19.05亿元，增长24.31%；完成全社会固定资产投资138亿元，增长19.2%；城镇居民人均可支配收入10530元，农民人均纯收入2800元，分别增长18%、9%。金融机构各项存款余额381.8亿元，增长16.4%，各项贷款余额210.8亿元，增长13.9%。商贸流通活跃，实现社会消费品零售总额128.6亿元，增长16.5%。非公有制经济快速发展，完成增加值175.5亿元，增长17%。招商引资取得重大进展，全年签约项目287个，其中内资项目268个，合同引进资金186亿元，实际到位55.4亿元；外资项目19个，总投资2.33亿美元，实际到位7000万美元。完成进出口总额6.5亿美元，增长14%，增速居全省前列。节能减排工作成效显著，规模工业企业万元增加值能耗下降9.91%、工业企业主要污染物排放达标率96.6%、工业企业环保“三同时”执行合格率100%，三项指标完成情况在全省排名第一，二氧化硫、化学需氧量削减率达到3%以上。县域经济竞相发展，活力增强，各县市区经济总量跃升，效益提高，冷水江市迈入全省经济十强县市前5名。

　　产业结构不断优化。一、二、三产业增加值分别为80亿元、213亿元、144亿元，分别增长5%、17.3%、13.1%，三次产业结构比为18.3：48.7：33，呈现出“二三一”产业结构特点。坚持以新型工业化为第一推动力，深化企业改革，加快技术创新，工业的主导地位更加突出。全市规模以上工业实现增加值167亿元，增长21%；实现利润22亿元，增长48%；实现利税58亿元，增长66.8%；工业经济效益综合指数达到200%。培育集群产业成效明显，重点培育的10大产业集群实现主营业收入477.24亿元，占全市规模工业主营业收入的82.97%。高新技术产业发展迅速，省级以上高新技术企业达到21家，完成产值110亿元，增长20%。“闪星牌”商标成为我市继涟钢“双菱牌”之后的第二个“中国驰名商标”，“闪星牌三氧化二锑”入选中国名牌产品，实现了我市中国名牌产品零的突破。园区工业增势强劲，完成工业增加值7亿元，增长50%。593家市县属国有企业改制基本到位，成功引进湖北宜化重组金信化工，企业重新焕发了生机与活力。农业结构调整步伐加快。全年粮食产量171.25万吨，增长0.8%；新增省市农业产业化龙头企业28家；生猪和草食动物、优质米、中药材、果蔬、楠竹五大产业不断走向产业化、规模化、品牌化；“永丰辣酱”获国家地理标志保护产品，“御唐叶”产品全部出口日本，“湘中黑牛”成为2008年北京奥运会专供产品。商贸流通、旅游、信息、金融等第三产业保持较快增长态势。“万村千乡”工程有序推进，春园商业步行街、五江国际建材城建成营运，沃尔玛春园店、五江店相继开业。旅游产业发展步伐加快，创建中国优秀旅游城市工作全面启动，全年旅游接待312.3万人次，实现旅游收入15.5亿元，分别增长34.7%、36.8%。

　　人居环境日益改善。坚持以人为本，加大建设投入，促进城乡互动，城乡面貌大为改观。娄底相继获得省级卫生城市、园林城市、文明城市和全国绿化模范城市荣誉后，又入选全国十大宜居城市。交通状况大为改善。全年完成交通固定资产投资8.57亿元，增长16.8%，二广高速、娄新高速基本完成前期准备工作，S312线涟源至新化段改建工程竣工，完成县到乡镇公路71.4公里、乡到村水泥路2248.3公里。城市基础承载能力和综合服务功能进一步增强。市本级开工建设项目44个，完成城市基础设施建设投资4.1亿元，新建改造城市道路20公里，垃圾填埋场投入使用，污水处理厂即将竣工，中心城区107条主要街道命名工作全部完成；中心城区绿地率32.6%、绿化覆盖率36.2%，全市森林覆盖率44.9%。县市城区与小城镇建设步伐加快，城镇化率达到34%。社会主义新农村建设加快推进，78个示范村建设取得阶段性成效，解决16.4万农村人口的安全饮水问题；库区移民后扶工作取得突破性进展，解决近33万人的连续20年的生产生活扶持问题。

　　社会事业稳步推进。着力推进创新型娄底建设，科技事业迅速发展，成功举办了全国农村科技合作组织发展论坛，双峰农村科技合作社模式在全国推广，我市被列为国家知识产权试点城市。以建设教育强市为契机，教育事业稳步发展，基础教育、职业教育、高等教育、成人教育均有新的提高，高考上线率连续5年全省第一，农村义务教育阶段学生学杂费全部免除。公共卫生体系进一步完善，重大传染病、地方病、职业病和突发性公共卫生事件得到有效控制，新建改造乡镇卫生院8所，新型农村合作医疗试点工作平稳推进，连续5年被评为全国无偿献血先进城市。文化新闻出版和体育事业蓬勃发展，市体育中心开工建设，广播电视村村通和有线数字电视建设工作稳步推进。8件实事全面完成，人民群众得到更多实惠。农村最低生活保障制度全面推行，新增农村低保98000人，新建农村敬老院13所、村级五保之家19所。劳动保障和就业体系进一步完善，新增社会养老保险参保人数13723人，全年新增城镇就业31200人，下岗失业人员再就业15956人，解决零就业家庭就业732人。经济适用房和廉租住房保障力度加大，新建经济适用房18036平方米，安排低收入家庭141户，新建廉租住房22584平方米，安置低保户376户。住房公积金归集资金3.8亿元，解决3500户家庭住房贷款3.3亿元。人口计生工作成效显著，低生育水平得到巩固，进入全省一类行列。生产、交通和消防安全管理进一步加强，安全生产形势进一步好转，亿元GDP生产安全事故死亡率下降8%，年内没有发生重特大安全事故。高度重视普法、信访、调解工作，依法严厉打击各种犯罪活动，确保了社会稳定。人民武装、工商、人事、物价、审计、统计、人防、食品药品监督、质量技术监督、对台、外事侨务、民族宗教、气象、地震、档案、老龄、残联、红十字会等各项工作均有新的进步。

　　政府自身建设不断加强。坚持重大决策和重大事项向人大及常委会报告、与政协协商，全年共办理市人大常委会交办函6件，全国、省、市人大代表议案建议158件和政协委员提案、政协建议案190件，办复率100%，满意率99.2%。坚持民主决策、科学决策，在决策前深入调研，决策中广泛听取意见，决策后加强督查落实，及时解决了全市经济社会发展中的一些重大问题。扎实推进政务公开,市县政务中心服务不断优化，办事程序不断规范，效率不断提高。大力推进村务、社区事务和厂务公开，基层民主建设不断加强。切实转变干部作风，坚决纠正行业不正之风，集中治理商业贿赂，严厉查处违法违纪案件，经济发展环境进一步优化，政风行风明显好转。

　　各位代表，一年来所取得的这些成绩，是省委、省政府正确领导、亲切关怀的结果，是市委统揽全局、科学决策的结果，是市人大和市政协加强监督、大力支持的结果，是全体人大代表、政协委员和全市人民团结奋斗、顽强拼搏的结果。在此，我代表市人民政府，向全市人民，向各民主党派、工商联和无党派人士，向工会、共青团、妇联等群众团体和离退休老同志，向驻娄解放军、武警官兵，向中央和省驻娄各单位，向所有关心支持娄底发展的各界人士和朋友，表示崇高的敬意和诚挚的感谢！

　　在充分肯定成绩的同时，我们也清醒地看到，我市经济社会发展中仍存在不少矛盾和困难，主要是：结构性矛盾依然突出，发展方式比较粗放；城乡发展不协调，县域经济发展不平衡；市县乡财政普遍紧张，收支矛盾比较突出；物价上涨偏快，通货膨胀压力增加；安全维稳形势不容乐观，影响社会稳定和谐的因素仍然较多；少数地方和部门法治意识与服务意识不强、办事效率不高、为政不廉现象依然存在，经济发展环境有待进一步优化，政府自身建设有待进一步加强。对此，我们一定高度重视，对面临的困难和问题始终保持清醒头脑，采取有效措施加以解决，绝不辜负全市人民的重托和期望。

　　二、2008年政府工作目标与总体要求

　　2008年，我们将迎来改革开放30周年。30年的改革发展特别是近几年来的后发赶超，使我市的经济社会发展活力大为增强。新的一年，我市的发展面临新的机遇和挑战。一方面，世界经济全球化、区域经济一体化进程加快，全球经济加快重组和国际产业加速转移，世界经济保持稳定增长，沿海地区向内地产业转移加速，地区间经济合作加强，我国工业化和城市化步伐加快、消费结构升级等，国内经济将继续保持较快增长态势，特别是中部崛起战略稳步推进、泛珠三角区域经济合作不断深化、长株潭 “两型社会”和“3+5”城市群建设全面启动，所有这些都为我市经济社会的加快发展提供了新的动力和平台。另一方面，我们更要看到，今后一段时期，中央把防止经济过热、防止明显通货膨胀作为宏观调控的首要任务，实行稳健的财政政策和从紧的货币政策，并将节能减排作为宏观调控的第三道闸门，同时，我市经济社会发展过程中结构、资金、项目、土地、环保等矛盾相互交织，更加突出，从而使我市的经济社会发展面临更多的压力和挑战。对此，我们必须增强忧患意识，敢于迎接挑战，善于抢抓机遇，立足娄底实际，按照人民要求，努力化解经济社会发展中的突出矛盾和问题，在加速赶超、跨越发展的新征途上乘势而上，奋勇争先，不断开创全市经济社会发展新局面。

　　2008年政府工作的总体要求是：认真贯彻党的十七大精神，高举中国特色社会主义伟大旗帜，深入贯彻落实科学发展观，坚持又好又快、好字优先，深化改革开放，推进自主创新，转变发展方式，加速“三化”进程，壮大经济总量，矢志后发赶超，加快富民强市，努力构建和谐娄底。

　　主要预期目标是：地区生产总值增长12.5%以上，其中第一产业增加值增长4%，第二产业增加值增长16%，工业增加值增长16.5%，第三产业增加值增长12%；全社会固定资产投资增长20%，利用外资增长12%以上；社会消费品零售总额增长16%，居民消费价格指数控制在105%以内；财政一般预算收入增长13%以上，城镇居民人均可支配收入增长10%，农民人均纯收入增长7%；新增城镇就业3万人，城镇登记失业率控制在4.5%以内；单位GDP能耗下降5%，化学需氧量减排2%，二氧化硫减排2%；亿元GDP生产安全事故死亡率下降8%；人口自然增长率控制在7‰以内。

　　要实现2008年政府工作目标，我们在具体工作中必须牢牢把握以下四点：

　　第一，必须坚持科学发展。以科学发展观统领经济社会发展全局，加快转变经济发展方式，加速实现后发赶超。遵循市场经济规律，充分发挥政府主导作用，积极创造公平竞争环境，推动经济社会协调发展。把项目建设作为促进市域经济发展的主要抓手，以大项目推动大发展，以好项目推动快发展。

　　第二，必须推进改革创新。坚持把改革创新作为推进经济社会发展的不竭动力。继续深化改革，扩大开放，不断推进观念创新、科技创新、制度创新、管理创新，努力提升经济发展的综合竞争力。

　　第三，必须加强“三基”工作。基础设施、基础产业、基础工作是突破市域经济发展薄弱环节、提升经济发展水平的关键。坚持把加强“三基”工作作为推动经济社会发展的重要举措来抓，不断夯实发展基础，增强发展后劲，促进我市经济社会加快发展、科学发展、和谐发展。

　　第四，必须注重改善民生。立足以人为本，把政府工作的出发点和落脚点真正放在为民谋利益上，着力解决人民群众最关心、最直接、最现实的利益问题，不断促进社会公平正义，让广大人民群众共享改革发展成果。

　　三、积极推动经济又好又快发展

　　认真贯彻党的十七大和中央、省委经济工作会议精神，主动应对国家宏观调控政策，深入推进改革创新，坚持把新型工业化作为第一推动力，加速推进农业产业化和新型城镇化，不断壮大县域经济，推动全市经济再上新台阶。

　　强力推进新型工业化。立足资源优势和产业基础，突出结构优化和自主创新，坚定不移地走新型工业化道路。坚持把培育产业集群作为推进新型工业化的重要途径来抓。积极引导和促进优势产业和骨干企业向集群方向发展，推进产业延伸和产业集聚，着力构筑和发展10大产业集群。全年建设重大工业项目27个，完成投资110亿元，重点抓好涟钢600万吨钢结构调整、冷钢二期300万吨钢结构调整、金电扩建二期工程、华润涟源煤电一体化工程、国产实业涟源水泥生产线、海螺双峰二期工程、海螺新化项目、涟源汇源120万吨焦化工程、安石集团城市煤气配套工程等项目建设。把培育产业集群与发展配套中小企业结合起来，形成优势产业、骨干企业与配套企业良性互动的工业发展格局。坚持把增强自主创新能力作为推进新型工业化的重要环节来抓。鼓励和支持有条件的企业建立研发机构，加大研发投入。加快利用先进技术特别是信息技术改造提升传统支柱产业，重点抓好涟钢CSP工艺生产高强度钢的研究开发和娄底德胜能源的氢氧燃料系列设备产业化开发。大力发展生物医药、新材料、先进制造、电子信息等高新技术产业，重点发展双峰农机、涟源煤机、新化电子陶瓷、冷水江耐火材料等产业，力争全年高新技术产值增长34%，达到130亿元。坚持把工业园区作为推进新型工业化的重要载体来抓。加大力度推进市经济开发区和各县市区工业园区建设。认真做好园区工业规划，完善园区体制机制。加强园区基础设施建设，切实提高园区的要素聚集和产业承载能力。确保用地、项目、资金适当向园区倾斜，促进工业园区更快发展。全市园区完成固定资产投资23亿元以上，实现工业增加值10亿元以上。加快市经济开发区第二工业园建设，着力打造薄板深加工产业基地，新增工业产值6亿元以上。坚持把发展非公有制经济作为推进新型工业化的重要支撑来抓。继续实施加快非公有制经济发展的“四个一”工程，完善发展非公有制经济服务体系，进一步加大引导和扶持力度，促进企业建立现代企业制度，完善法人治理结构，做大做强非公有制经济。力争全年非公有制经济增加值增长17%，达到205.3亿元，税收增长20%，达到16.6亿元。

　　深入推进新农村建设。以深化农村综合改革为抓手，积极稳妥推进基层农业技术推广体系、集体林权制度、农村小型水库产权制度、农业保险试点工作等各项改革，全面落实各项支农惠农政策，有效遏制和化解乡村债务，稳步发展农村经济，巩固和强化农业基础地位。切实提高农业综合生产能力。继续推进电网改造、通达通畅、农田水利、乡村清洁等农村基础设施建设，重点抓好新化、涟源的地方电网改造工程，尽快实现“同网同价”，确保农村生产生活的用电需求。坚持把“安全工程”放在农业农村发展的突出位置，切实抓好饮水安全、生态安全、防汛抗旱安全、动植物疫病防治安全。推广双峰建设农村科技合作社的经验，加快发展农村专业合作组织，推进农业科技进村入户，逐步推动农业信息化发展。加快发展现代农业。稳定发展粮食生产，确保粮食安全。认真抓好湘中粮食物流园等优粮工程和大型商品粮基地项目的申报实施工作，稳步发展“菜篮子”工程。加快发展特色农业和现代种养业，重点支持天华牧业、百雄堂、同星米业、庆阳牧业、湖南景龙等省市级龙头企业的发展，力争全年农产品加工产值增长20%以上，达到38.4亿元。大力发展劳务经济和技能经济。培育有文化、懂技术、会经营的新型农民，完善劳务输出网络，维护务工人员合法权益，全年培训农民26万人次，新增农村劳动力转移就业2万人以上。

　　加快推进新型城镇化。主动对接“3+5”城市群发展战略，积极融入长株潭“两型社会”建设发展新平台，明确城市发展定位，促进城乡良性互动，进一步巩固和提高娄底在全省乃至全国的能源原材料基地、区域性交通枢纽和全国十大宜居城市地位。加快基础设施建设，着力提高城镇化水平。切实抓好“一点两线”城镇带和重点小城镇建设，重点抓好中心城区和各县市城区扩容提质。中心城区继续坚持“扩容提质、完善功能、改善环境、提升品位”的方针，加快推进城市环线建设，重点加快吉星路建设进度，全面启动株山公园、甘桂路等重大项目，加快推进市经济开发区、城南新区、娄星经济园区配套道路建设，继续加强小街小巷配套改造，完成污水处理厂和垃圾中转站项目。坚持高起点、高质量，加快建设“娄底—涟源—冷水江—新化”城镇带和“娄底—双峰”城镇带，着力打造特色鲜明、优势突出的“湘中经济走廊”。以强化功能、彰显个性、集聚人口为发展方向，下大力气抓好县市城区和重点小城镇建设。计划全年市政公用设施建设投资8亿元，新扩建城市道路20公里，新增供水管道40公里，力争城镇化率达到36%。坚持建管并重，切实提升城市综合管理水平。加强规划管理，严格规划执法，维护规划权威。运用法律、行政、市场、科技等多种手段，理顺体制，创新模式，加强城市综合管理。强化交通秩序、环卫设施、园林绿化等城市基础管理，进一步完善城市管理的长效机制，推进城市管理规范化，提升城市品位，优化人居环境。坚持把经济发展作为城镇化的重要支撑，加快发展县域经济。充分发挥城镇的聚集与辐射作用，明确功能分区，规划产业布局，以产业支撑城镇功能，以城镇功能促进产业发展。以县市城区为龙头，以城镇化建设为载体，进一步落实县域经济发展的各项措施，加大政策支持和有效投入，引导各县市区依托资源和优势，加快发展，增强竞争能力，提高发展水平。

　　大力发展开放型经济和旅游产业。坚持以改革开放为动力，不断创新思想观念和体制机制，全方位推进对外开放，壮大发展旅游产业，加快发展现代物流、商务服务、信息服务、科技服务、金融服务等生产性服务业，努力实现开放型经济发展的新突破。加快发展开放型经济。全力抓好招商引资，重点引进世界500强、国内上市公司、大型企业、港澳台企业等战略投资者，同时抓好招商项目的跟踪和落实，切实提高合同履约率。培育和壮大龙头出口企业，进一步调整和优化进出口产品结构，努力扩大娄底产品的市场占有率。改善外贸经济基础环境，争取年内设立娄底铁路口岸，推动外贸经济加快发展。壮大发展旅游产业。充分发挥旅游资源的优势，从更高层次、以更宽视野谋划和推动旅游产业发展，下大力气将旅游产业逐步培育成为支柱产业。大力实施旅游带动战略，以创建中国优秀旅游城市为总揽，扎实抓好创优各项具体工作，加快旅游交通、旅游产品和管理服务三大体系建设，做大做强旅游产业。坚持优先发展旅游交通，加快景区基础设施和其他配套设施建设。进一步整合旅游资源，优化旅游线路，打造旅游精品，重点培育富厚堂、紫鹊界秦人梯田、梅山龙宫、波月洞、大熊山国家森林公园、湄江风景区、龙山华夏中药文化园等旅游品牌。加快发展复合型旅游，推动过境观光向休闲、度假旅游发展，提高旅游综合效益。积极发展生产性服务业。立足区位和交通优势，加快发展现代物流等生产性服务业，抓好一批专业物流基地建设，培育一批竞争力较强的专业市场，重点引导钢铁、建材等优势产业物流市场加快发展。积极发展金融、保险、证券和咨询、法律、会计、信息等中介服务业，加快运用现代服务技术和经营方式改造提升商贸、餐饮、娱乐等行业，优化服务业结构，提升服务业水平。

　　有效推动投资和消费合理增长。进一步优化投资结构，扩大消费需求，努力促进投资和消费合理增长。突出抓好重大项目建设。计划全年建设重大项目60个，其中新开工18个，完成投资146亿元。基础设施方面，集中力量抓好高速公路和国道、省道等干线公路建设，重点抓好二广高速娄底段、娄新高速、G207线、S210线等项目的开工建设，力争S209线年内开工、娄底汽车南站尽快投入使用；抓好铁路改造工程，加快娄底火车站到达场改扩建工程。社会发展方面，加快市体育中心、星星实验学校和湖南人文科技学院、娄底职业技术学院、娄底卫校升专改扩建等项目的建设进度，开工建设市中心医院住院大楼和市中医院综合大楼等新建项目。在抓好在建项目的同时，认真做好新化火电厂、益娄衡高速、冷水江火车东站扩能改造、天然气管输工程等17个重大项目的前期工作，积极策划和储备一批符合国家产业政策、产业关联度高、带动能力强的重大项目。积极拓宽筹融资渠道。认真研究和把握国家产业政策导向，积极争取国家和省里的项目、资金支持，特别是在事关全局的公路、能源、矿区治理等基础性重大项目方面要加大向上争取扶持的力度。不断创新融资方式，加大市场融资力度，引导和促进民间投资。制定优惠政策，支持和推动有条件的企业尽快上市。深入开展金融安全区创建工作，加强企业信用体系和担保体系建设，扩大银地、银企联合，提高金融机构信贷投放率。努力扩大城乡消费需求。认真落实增加居民收入的各项政策，努力提高低收入者的收入水平和消费能力。合理引导住房、汽车等消费，积极培育消费热点，促进居民消费结构升级。加强农村市场体系建设，有序推进“万村千乡”工程和“新网”工程，积极引导连锁店和超市向农村延伸，全面拓宽农村消费市场。

　　四、大力发展先进文化

发展先进文化，建设文化强市，既是我市加速赶超的重要内容，也是广大人民群众的迫切愿望。坚持百花齐放、百家争鸣的文化建设方针，按照“办好文化事业，壮大文化产业”的思路，大力加强文化建设，用先进文化推动全市政治、经济、社会的全面协调发展。

　　着力培育健康文明的社会风尚。坚持马克思主义在意识形态领域的指导地位，着力建设社会主义核心价值体系，用中国特色社会主义共同理想凝聚全市人民，弘扬“坚韧、勤奋、尚德、自强”的娄底精神，为建设小康、和谐娄底奠定坚实的思想基础。大力开展爱国主义、集体主义、社会主义教育，继续深化社会主义荣辱观教育，引导人民树立正确的世界观、人生观和价值观。深入开展社会公德、职业道德、家庭美德、个人品德教育，努力形成诚信为本、平等友善、遵守公德、文明礼貌的社会风尚，努力建设诚信娄底。持续推进创建文明城市、文明村镇、文明社区、文明行业、文明单位、文明窗口、文明家庭活动，不断提高全社会的文明程度。

　　加快发展文化事业。加快国有经营性文化单位和公益性文化事业单位的改革步伐，理顺产权关系，不断激发内部活力，增强服务能力。尽快启动文化中心建设，深入实施公共文化惠民工程、文化信息资源共享工程、广播电视村村通和有线数字电视工程，逐步推进 “农家书屋”建设，年内完成11个乡镇文化站建设任务，建立覆盖城乡的公共文化服务体系，让广大群众共享文化发展成果。积极开展彰显“青春娄底”个性的广场文化、社区文化等群众性文化活动，丰富人民群众的文化生活，促进人的全面发展。大力开展文艺、图书、电影等文化下基层活动，不断满足基层群众日益增长的文化需求。建立文化精品奖励机制，积极引进专业人才，提高文化原创能力。进一步做好古城、古镇、古街和名人故居、历史遗址、文物的保护与开发工作，切实做好非物质文化遗产普查工作，编辑出版反映娄底人文特色的作品，不断扩大娄底在国内外的影响力。切实加强文化市场监管，深入开展“扫黄打非”和网吧整治工作，优化文化发展环境。

　　不断壮大文化产业。树立抓文化产业也是抓经济建设的理念，不断完善和落实文化产业扶持政策，解放和发展文化生产力。实施文化品牌战略，着力搞好全市文化产业规划，重点培育以蚩尤文化、梅山文化为代表的区域文化品牌，以曾国藩、蔡和森、陈天华、罗盛教为代表的历史人物文化品牌，以锡矿山、涟钢为代表的工业文化品牌，不断扩大全国武术之乡、中华诗词之乡、中国书画之乡、中国女杰之乡的影响力。进一步整合全市文化资源，着力培育成长性好、潜力大的文化企业，促进文化资源向经济资源转变。重点支持报刊、广播电视、新闻出版、网络媒体等文化产业加快发展，继续扶持体育、影视、演艺、娱乐、饮食等与文化相关的第三产业和会展业、快乐购物等新兴文化产业发展，不断提高文化产业在GDP中的贡献率。

　　五、努力构建和谐娄底

　　在加快经济发展的同时，要积极推动以改善民生为重点的社会建设，促进经济社会协调发展，努力构建和谐娄底。

　　着力保障和改善民生。按照“学有所教、劳有所得、病有所医、老有所养、住有所居”的总体要求，把民生问题摆在更加突出的位置，进一步加大财政投入力度，继续办好8件实事，让老百姓充分享受到改革与发展的成果。坚持教育优先发展不动摇，加快教育强市建设步伐，突出抓好学前三年教育，均衡推进义务教育，基本普及高中教育，全面推行城乡义务教育经费保障机制改革，着力解决进城务工人员子女上学难问题，继续规范教育收费，坚决查处教育乱收费行为，逐步化解农村“普九”债务，努力办好让人民满意的教育。深化卫生医疗体制改革，完善公共卫生服务，推进农村新型合作医疗和城市社区卫生医疗服务体系建设，继续开展医疗卫生单位的行风评议工作，加强医疗市场管理，创建“平安医院”，构建和谐医患关系。认真做好人口与计划生育工作，落实各项计生奖励和救助制度，有效遏制出生性别比失衡，积极发挥计生协会作用，稳定低生育水平。大力推进科技进步，组织实施重大科技专项，加快创新型娄底建设步伐。大力实施人才强市战略，加快湘中人才市场建设，着力培养各类人才，为加快发展提供强有力的人才支撑。完善市场就业机制，加强就业指导和服务，鼓励自主创业和自谋职业，促进以创业带动就业，千方百计扩大城乡就业，重点解决零就业家庭就业问题，切实加强军队转业干部、复退人员的安置和高校毕业生的就业指导服务工作，认真做好下岗失业人员再就业、残疾人就业工作。加快完善社会保障体系，以民营企业职工、农民工、个体工商户和灵活就业人员为重点，进一步扩大养老保险、医疗保险、失业保险和工伤保险的覆盖面，启动城镇居民基本医疗保险工作，完善农村最低生活保障制度和社会救助体系，加快推进城镇经济适用房和城镇低收入家庭廉租房建设。继续加大扶贫开发力度，认真落实库区移民后期扶持政策，着力解决特困家庭和弱势群体生活困难等突出问题。加强政府对市场的调控力度，努力保持物价总体水平基本稳定。切实维护老年人、妇女儿童和残疾人的合法权益。继续统筹做好审计、统计、外事侨务、民族宗教、贸促、对台、地方志、档案、气象、地震、红十字会等工作，推动社会全面进步。

　　大力建设生态文明。合理利用资源，优化产业发展，强化环境保护，努力走一条青山绿水与富民产业共存共荣的绿色发展之路，着力构建环境保护与经济增长协调发展的生态娄底。坚持开发与节约并举、节约优先，推进资源节约和综合利用。严格实行节能减排工作责任制和问责制，强化节能减排工作考核，加强重点领域、重点行业和重点企业的节能减排工作，依法淘汰浪费资源和污染环境的小煤矿、小焦炭、小水泥、小冶金等落后生产能力。加大节能环保投入，重点抓好工业污染整治和涟邵矿区采煤沉陷区、冷水江矿区采矿沉陷区治理，强化对资水、涟水、孙水、湄水等主要河流排污控制，加强对饮用水源的保护，加快推进4个县市城区的垃圾填埋场建设。落实新上项目节能环保评估制度，严格规范环保审批程序，严把能耗增加和污染排放源头关，对违反节能减排规定的行为，严厉查处，绝不姑息。大力推行清洁生产，加快发展循环经济，促进资源循环利用。认真落实耕地保护责任制，严格控制建设占地规模
